# Genetic Engineering and Human Mental Ecology: Interlocking Effects and Educational Considerations

**DOI:** 10.1007/s12304-017-9286-7

**Published:** 2017-03-28

**Authors:** Ramsey Affifi

**Affiliations:** Education, Teaching and Leadership (ETL), Moray House School of Education, University of Edinburgh, Edinburgh, EH8 8AQ UK

**Keywords:** Genetic engineering education, Genetics education, Second-order cybernetics, Sustainability, Gregory Bateson, Mental ecology, Mental ecology, Ecology of mind, Hubris, Reverence

## Abstract

This paper describes some likely semiotic consequences of genetic engineering on what Gregory Bateson has called “the mental ecology” (1979) of future humans, consequences that are less often raised in discussions surrounding the safety of GMOs (genetically modified organisms). The effects are as follows: an increased 1) habituation to the presence of GMOs in the environment, 2) normalization of empirically false assumptions grounding genetic reductionism, 3) acceptance that humans are capable and entitled to decide what constitutes an evolutionary improvement for a species, 4) perception that the main source of creativity and problem solving in the biosphere is anthropogenic. Though there are some tensions between them, these effects tend to produce self-validating webs of ideas, actions, and environments, which may reinforce destructive habits of thought. Humans are unlikely to safely develop genetic technologies without confronting these escalating processes directly. Intervening in this mental ecology presents distinct challenges for educators, as will be discussed.

## Introduction

Intervening directly in genomes, by inserting, creating, or altering the function of genetic material, is often seen as the basis of the next industrial revolution (Church and Regis 2012; Dyson 2007). Promised is a new economy unlimited in capacity to further human health, comfort, and prosperity. The actual and potential applications of such genetic interventions are tremendous, as much for the life sciences as for commerce more generally. Genetic engineering has already been used to mass-produce cheap insulin for diabetics, human growth hormones, antibodies, anti-hemophilic factors, and vaccines. It may fruitfully produce biofuels, remediate contaminated areas, and generate bacteria that can detect toxins such as arsenic in water. Furthermore, recent genetic engineering procedures, such as CRISPR cas9 gene editing, are much easier and powerful in scope, and are widely recognized as an industry “game changer” in what is already a game changing industry. Regardless of whether such advancements live up to their hype, these moderate gains already present society with powerful tools.

Nevertheless, genetic technologies have faced considerable backlash from environmentalists and food and health advocates, which has probably slowed commercialization in some sectors.^1^ Those concerned often cite “potential risks” and call for the “precautionary principle”, which maintains that humans should wait until a new technology is reasonably shown to be safe before using or commercializing it (see O'Riordan and Cameron 1994). Such a requirement seem untenable to proponents because technology (or *any activity* for that matter) has always proceeded with unknown risks. Genetic engineering is no different on this account, and should not therefore require more regulation or scrutiny than other human activities. As is often pointed out, it cannot be proven that we will not be hit by a car when crossing the street but this does not (nor should it) prevent us from crossing. The precautionary principle is probably inherently self-contradictory because *not doing something* often has its own risks and would require evidence prior to its own implementation (Mandal and Gathii 2006). As agricultural economist Jayson Lusk expressed it: “There are risks with these technologies, there are risks with every technology. There are also risks with not approving these technologies” (CBC News 2015).

Whether and in what conditions the precautionary principle is tenable is an important discussion, but it is not the only way to think about genetic technologies. The approach adopted in this paper does not appeal to threatening unknowns. It instead articulates some semiotic effects likely to occur through the increasing use of engineering practices and metaphors applied to modifying life. I hope to show that thinking about other organisms in engineering terms leads to human activities that profoundly transforms our sense of what it means to be “human” and our understanding of “nature.” This transformation entails major risks that cannot be simply cast aside as anti-scientific doomsday prophesying. As we shall see, the unreflective normalization of genetic engineering leads directly to the perpetual re-creation of increasingly vast parts of the biological world, at once exacerbating an ecological and a “spiritual” crisis. This is because at the same time as increasing numbers of novel organisms are introduced into already degraded and resilience-compromised ecosystems, our experience of natural beauty, complexity, and creativity is progressively replaced with *signs* of human ingenuity, purposefulness, and, when things go wrong, error. While a concern with the semiosis of humans and nature seems but philosophical extravagance, I maintain that a sense of wonder and appreciation of the nonhuman world is not a bourgeois or romantic fantasy but an essential part of experience that we must cherish and nurture. This does not necessarily mean banning all applications of genetic engineering, but it does imply developing a strong understanding of what grounds and supports our lives, physically, emotionally, spiritually, and aesthetically, and the courage to allow these factors to inform our science policy and practice. Science and environmental educators need to be aware of and sensitive to these possible consequences and to take the necessary steps to prevent undesirable effects.^2^

The structure of the paper is as follows. I first describe a framework developed by semiotician and cybernetician Gregory Bateson known as “mental ecology” (1972; 1979), which is fruitful for understanding how metaphors (such as “engineering” life) influence and spread. I then employ this framework to describe some effects of genetic engineering that interlock in potentially harmful ways. For the scope of this paper, I focus on four forms of habituation that are already underway but are expected to sharply increase. These are habituation to 1) GMOs as a normal presence in our environments, 2) the idea that organisms are programmed by atomistic pieces of code, 3) the sense that gene-level changes to humans and other species are improvements, and 4) the sense that creativity is something uniquely and ubiquitously human. These forms of habituation occur independently of whether or not GMOs are harmful or beneficial in any *particular* application. As such, they necessarily slip past current regulatory schemes. If such aggregating habituations are likely to lead to dangerous outcomes, we must examine what are our possibilities for averting such harm. Science educators at all levels play an important role in addressing this challenge and specific educational implications will be discussed in the final section of the paper.

## Batesonian Minds

This paper is concerned foremost with the effects of genetic engineering on *how we think*, how semiotic shifts alter our actions and thereby our environments, and what considerations can be made by educators wishing to engage in this process. I am guided here by a Batesonian approach to thinking about problematic semiotic trends and dynamics associated with genetic engineering. By “Batesonian,” I mean that it aligns with a general pattern of thought that Gregory Bateson struggled to develop during his lifetime for thinking about ecological phenomena as diverse as biological ecosystems, addiction, disease, and human relationships. After briefly sketching this out, I will apply some of his ecological thinking to helping clarify the dynamics of semiosis in the age of genetic engineering.

Bateson defined “mind” functionally as a process that occurs given certain types of organization and he sought to articulate the basic aspects of that organization. The details of his proposal do not matter for this paper (see Bateson 1979), but what is essential here is that he considered mind not to operate in the brain or even in the body (as Freudians had extended it) but rather to be located in a broader or extended system that he described as “organism plus environment.” An animal, for example, is continuously engaging its “external” world by picking up information through its senses, which gets translated by sensory organs, creating new information for nerve cells, and so on, until it reaches the brain and gradually makes its way to the motor organs to create some sort of change in the relationship between the organism and that stimulus in its environment. What is going on at each stage is a response to *difference* (i.e. the stimulus in the external environment was created by distinguishing it against a context, which thereby elicited a difference in the sensory organ that similarly spurred another difference in the nerve cell it communicated with, and so on, ending with the organism creating some difference in its relationship with its environment), so minds can be considered as systems set up to respond to and circulate *difference*. And, because the system is set up in a circular way (since the motor changes alter the environment that the organism is capable of responding to, re-constitute the stimulus, and change it in the next cycle), Bateson’s basic characterization is that a mind is a *recursive* system that constitutes and responds to differences. Finally, for Bateson, a mind is also hierarchically organized. It is not a single recursive system, but a recursive system regulated by other recursive systems that are responding to differences *in it* (this is why Bateson explained that organisms do not merely “learn” but “learn to learn” and sometimes even “learn to learn to learn” (see Bateson’s discussion on levels of learning (1972: 279–308)).

Mind, in a Batesonian sense, is ubiquitous across the biological world, from single cells to multicellular organisms, to ecosystems. As recursive systems, some machines (such as thermostats) are also mental, though insofar as they have no hierarchy of recursion (and therefore no learning) they are better thought of as proto-mental. Importantly, for Bateson mind is not necessarily “conscious,” in the self-referential sense of being aware *that* it is aware.^3^

For Bateson, minds can be “healthy” or “pathological” depending on how their ongoing responsiveness to information affects their relationship with their larger interdependencies. Pathology occurs when a mind responds to its environment by making (or failing to make) distinctions resulting in the reduced viability of the “organism plus environment” system. Because these distinctions are epistemological, pathology is considered an “error.” When errors circulate through recursive systems they can become self-validating: the organism modifies its environment based on whatever distinctions it has made and the errors get reified (i.e. the environment now embeds these errors physically into itself, which the organism interprets as evidence that its interpretation is valid). This is why in an important article on alcoholism, Bateson (1972) describes addiction as an “epistemological” problem created and exacerbated by an *incorrect* way of understanding oneself and one’s capacities, an error which gets re-enforced by how a person and her physical and social environments reciprocally interlock as the disease progresses.

Such self-validating processes are called “positive” or “runaway” feedback loops. Positive feedback loops are not sustainable in ecosystems because they increase a subset of variables at the expense of other essential variables. Such subsystems insulate themselves against buffering, so rapid growth follows but so does disruption of the surrounding system, leading to loss of function, versatility, or resilience of the system as a whole. Because the larger context of the runaway subsystem cannot support indefinite expansion, the subsystem is eventually either dampened by the larger system (like how the population growth of a herbivore is subsequently buffered by the growth of its predator) or it destroys the larger system entirely (such as some cancers). It is for this reason that Bateson considered uncontrolled positive feedback loops as pathological. And “health” is conversely related to attenuating or reversing runaway feedback loops occurring in mental systems, which is perhaps why healthy ecosystems (and people) are said to be “balanced.” In healthy mental systems various recursive feedback loops govern one another preventing any from ever-escalating positive feedback. When discussing aspects and outcomes of positive and negative feedback loops, Bateson is often explicitly concerned with the semiotic dynamics of human mental ecologies. This is evident, for example in his discussion of the mutual reinforcement of hubris and technology (Bateson 1972). Bateson’s concern with semiosis is broader and more systemic than Peirce who tends to be concerned with particular rather than global semiotic events.

The purpose of this essay is twofold: 1) to uncover how the engineering metaphor applied to living systems leads to several positive feedback loops, and 2) to examine how educators can assist in balancing these runaways. As we shall see, particular positive feedback loops emerge that reinforce problematic ways of understanding and acting towards other species.^4^ Insofar as these feedback loops lose flexibility and insulate against correction, they are pathological for humans and other species involved. I examine four such interlocking loops in this paper but also identify some limitations to my analysis. We ought to develop a society that can scrutinize genetic engineering carefully so I discuss pedagogical issues involved with meeting this end in the final section of the paper.

## Habituation to GMOs in our Environment

The first feedback loop associated with the development of GMOs discussed is so simple that it seems to hardly deserve investigation. It is this: the more GMOs are present in our environment, the more people get used to them being there, and the less likely they will be to remain sensitive to their presence. Organisms habituate to what they are immersed in, rendering constant inputs or stimuli a part of their background context. Novel things settle into the subconscious, the body and mind develop habits to deal with them, and thinking moves on to handle other things. From a Batesonian perspective, habituation to accumulating factors means that difference (or information) is not circulating within the mental system, not effecting perceptual-motor adjustments, and what could drive action becomes increasingly treated as “context”. Bateson recognized that this process was inherent in our capacity to learn. For example, it is only because humans are able to see similar things as identical that we can form classes or categories, which we use to make distinctions. While this produces conceptual blocks that enable thinking, it is a double-edged sword with clear shortcomings: “precisely because the mind can receive news only of difference, there is difficulty in discriminating between a *slow change* and a *state*. There is necessarily a threshold gradient below which gradient cannot be perceived” (Bateson 1979: 98). The educational challenge is therefore a semiotic one: to help students develop skills to treat individual cases *as such* when needed and to circumvent the tendency to categorize and overlook difference.

Habituation is self-validating. Or, as essayist George Monbiot puts it in the context of the ecological crisis in general, “with each subtle intensification, the baseline of normality shifts” (2015). Once some GMOs are accepted in our environment, their precedence facilitates the introduction of others. This happens on a microscale (for example, most new agricultural GMOs that have been approved since the public’s initial swell of concern in the late 1990s have been new varieties of species already genetically engineered (such as corn, soy, and cotton products)). But it also happens on a broader scale, as each new GMO paves the way for the likelihood of further GMOs, with the upcoming approval of GMO salmon in the United States expediting the approval of other genetically engineered animals, and so on.^5^

Currently, most GMOs involve the introduction of relatively little transgenic or edited DNA, a point continuously identified by proponents (for example, see Folta 2012). However, what needs to be critically evaluated is the cumulative effect microscale interventions have on the normalization of the technology, and in particular on shifting public acceptance with respect to living in a genetically engineered world. Once accepted, there is only an incremental difference between the insertion or editing of 1–3 genes and the modification of 5–10 genes, or between engineering a few domesticated food crops and engineering wild species writ large. Meanwhile, genetic interventions continue to become increasingly powerful. For instance, CRISPR-Cas9 technology now enables scientists to remove, insert, and modify genetic material much more easily than in the past, when trial-and-error insertions through firing genes into DNA, or inducing infections using viral or bacterial components as vectors, was common (Pennisi 2013). Chinese scientists recently used CRISPR editing technology to modify nonviable human embryos (Liang et al. 2015), and the National Academy of Sciences (2017) has recently recommended human gene editing for certain medical treatments. Further, with “gene drives” scientists can now seed populations of organisms with small quantities of genetically altered organisms produced to ensure the spread of the novel DNA material regardless of whether it confers a benefit or a liability on the organisms (Oye et al. 2014). That these ideas do not bat an eye now indicate that Batesonian shifting baseline effects are well underway.

There is only a continuous series of incremental differences between single-gene deletions and biotech entrepreneur Craig Venter’s age of “digital life” (2013), an anticipated stage when humans will purportedly be able to accelerate biological evolution up to the same rate as social evolution – which basically means transforming life at the rate of the development of thought itself.^6^ Unless the tendency towards habituation is explicitly addressed by educators, the acceptance of the technology will likely increase nonlinearly as the most fundamental ethical or existential questions (to accept genetic intervention or not) recede into the background through the acceptance of the initial GMO crops. An additional factor in this mental ecology is that precedence-setting is such a powerful process in human mental ecologies that it is sometimes considered explicitly in pedagogical recommendations aimed at fostering societal acceptance of genetic engineering. Freeman Dyson (2007) has us imagine that debate about biotechnology could be greatly attenuated if educators designed “biotech games” for children so that they could “intimately” interact with GMOs by growing up modifying other organisms for the sake of curiosity or competition. The same argument is put forward by Biofortified (Coatney 2013), which claims that do-it-yourself (DIY) biotech will push genetic engineering into public acceptance.

Once considered a powerful practice reserved for curing disease, genetic engineering has now been taken up by artists (who are often funded by university biology departments). For the purposes of provocation or beauty, the world now has Eduardo Kac’s rabbit engineered to glow in the dark, and the “cactus project,” a cactus engineered to produce human hair instead of thorns. There are now dozens of organizations and centres devoted to exploring transgenic organisms as art. At the same time, synthetic biology projects are now fundraising through online crowdsourcing websites such as Kickstarter, and do-it-yourself GMO organizations (such as Biocurious and DIYBIO) are setting up “community biology” groups for collaboratively participating in, and sharing experiences about genetically engineering other species. In addition, an international competition called iGEM has groups of high school and college students across the world compete against each other for yearly awards honouring the most interesting or innovative transgenic designs and biotechnologies. While some of these projects may serve vital human needs, as a whole they indicate an engineering attitude towards other organisms employed for increasingly frivolous purposes.

Is this normalization trend problematic? Clearly many biologists think not, but it is important to consider what is at stake. The invitation is to clearly understand what we are becoming accustomed to in our mental ecologies as well as the consequences of this learning process. In the remainder of this section and in those following it, I shall explore this question in greater detail.

Habituation to GMOs is clearly not habituation to a purely physical environmental novelty, such as occurs to a change of temperature. It is unlikely that most of us would recognize most GMOs if encountered untold. Instead, it is at once perceptual and conceptual because it involves becoming accustomed to a set of ideas as to what GMOs (and life in general, and we as agents) are. In the most general sense, through the increased presence of GMOs, people become accustomed to GMOs not simply as physical entities, but also as ideas, as complex discursive entities with growing, yet competing understandings, with accompanying sets of attitudes, including ranges of possible practices and posit-able futures, either of progress or doom. As the number of ways in which we think about and articulate what GMOs “are” increases, we get habituated to the notion that this very discussion or debate itself is “normal.” GMOs become normalized as a polarizing issue (like say, the abortion debate), and in so being categorized, thereafter constrain the responsivity of people to their existence. Normalization produces a relationality *between* attitudes that tends to produce a pattern of social relationships rather than precise behaviours.

In any case, the normalization of discourse is the most significant reason why there is no “going back” to the pre-GMO days. Even if people stopped producing GMOs entirely, the patterns of logic developed in articulating their presence and possibilities has already inflected subsequent thinking. For example, people increasingly think that “genetic modification” is something we have been doing “all along”, as they retroactively reconceptualise the process of selective breeding under this new frame. Domesticated animals are now commonly reconceived as artefactual because their past was marked by their being bred by or for us. This is a common bait-and-switch manoeuver by which humans make sense of their worlds. It is why Habermas argued that the “floodgate” or “slippery slope” argument is, in the cases of genetic engineering, valid: “accidental precedents and inconspicuous practices which [...] have become ingrained through normalization are retrospectively exploited, by those lobbying for genetic engineering and biotechnology, in order to shrug off moral misgivings as ‘too late’” (Habermas 2003: 19). Hence, we must learn to imagine and confront the semiotic evolution of possible technologies and their cumulative effects on our mental ecologies.

The problem is not simply that there are powerful lobby groups behind this retroactive reconceptualization, as Habermas has suggested.^7^ This retrofitting process is expected by the manner mental ecologies incorporate novelty. In the economy of mental ecologies, ideas that survive repeated use are more likely to be re-used (Bateson 1972: 509). Engineering metaphors, once applied to life, eventually come to be used in understanding not only our developing interactions with other organisms but our past and future interactions with them as well. In mental ecosystems, subsuming novelty under existing concepts is economical and necessary. As necessary as it is, without the complementary capacity to uncover and insulate against the effects of deleterious mental trends, we risk exacerbating these positive feedback loops. As mentioned, Bateson was aware that positive feedback loops are not sustainable because they tend to propagate a subset of interactants at the expense of a web of relations that these interactants depend upon. It will clearly be less painful for humans to buffer their activities through anticipating and avoiding systemic disturbance than letting the system as a whole buffer these activities later on. Education is critical to this process.

In the following sections, I will deal with more specific aspects of what is normalized laterally beyond GMOs as a set of concepts and discussions. However, as such an object of discourse, the term GMO establishes the context for the other forms of feedback discussed in this paper.

## Spreading the Doctrine of Genetic Reductionism

In philosophy of science, there are many varieties of ontological, epistemological, and methodological reductionism. Ontological reductionism is the position that any higher level property is but the sum of underlying interactions on some lower level. Methodological reductionism asserts that reductionist explanatory strategies and research approaches are the most effective for generating scientific knowledge. Epistemological reductionism understands reduction as essential to the meaning and process of knowing. Most lay attempts to describe the relationship between genes and organisms appeal to some form of ontological reduction, even though many scientists (when pressed on the matter) will agree that genetic reduction is at best methodological.^8^

For instance, assumptions about ontological reduction are apparent in how companies and the media describe drought resistant crops created by taking genes “for” drought resistance and putting them in new plants. Despite this shorthand way of speaking, a very different ontology has emerged about the relationship between genes, organisms, and environments. It is well-recognized that the informational value of genes is determined and continually readjusted by the organism itself, highlighting the fact that “causality” is distributed across various levels and scales in what is also a recursive system (Griffiths and Gray 1994; Oyama 2000). In other words, the informational value of genes and any other resources is determined by the ongoing semiosis of the developing organism (Bruni 2008; Affifi 2016), and ontogenetic development is itself mental in a Batesonian sense. With the discoveries of pleiotropy and epistasis,^9^ cracks in the reductionist paradigm emerged even before the rise of molecular biology, but the full extent of the interdependency and flexible adaptivity of the genome has really come to light in the past 10 years, with the discovery of hundreds of modes of chromatin remodelling, alternative splicings, DNA editing, RNA censoring, complex cis-regulating feedback networks, post-translational protein modification, etc. – all processes continually responding and re-responding to ongoing signalling changes occurring from within the cell and from outside it (Seo and Lee 2004; Griffiths and Stotz 2013; Jablonka and Lamb 1995; Shapiro 2011).^10^ In Batesonian terms, this means that the organism is able to adjust the meaning of a gene to its context, a hierarchical mental process which evidences “learning to learn” (1972).

It is ironic that twenty-first century genomics has discovered that the relationship between a gene and a phenotypic trait is more complex than we could possibly have imagined, and yet this realization is paralleled by a massive expansion of biotech crops and technologies. The incommensurability between knowledge (which crucially includes knowledge of the current limits of our knowledge) and practice is often brushed aside because much of genetics research is funded or partially funded by industry. As a result, an increasing rift is appearing between how geneticists understand the genome and how it is explained to the layman. Although geneticists are aware that it is rare for a gene to have a single and context-independent function, biotech companies often rely on just this sort of explanation when presenting their technology to the public. Biotech companies do not say “we inserted a gene which usually (but not necessarily) does X in species Y (along with an indefinite number of other things) into species Z, where it may or may not perform X in a certain set of contexts but also will likely do a bunch of unexpected things in its new cellular and genomic context.” Rather, a simplistic story is told: “the gene that makes a firefly luminescent is inserted into the *Arabidopsis* plant, and the product is a plant that now glows in the dark.” With the phenotype and the environment now devoid of contribution, the way in which all are connected recursively as a Batesonian mind is hidden from view.^11^ This is a typical consequence of forgetting that a methodological reduction is methodological and not ontological. The consequences however are vast.

There is a spectrum between those genes that are tightly and functionally integrated with other genes (and regulatory DNA) and genes which are quite modular and (more or less) functionally discrete. To be more specific, whether genes have interactional effects with one another or not is dependent on what West-Eberhard calls “developmental switches” (2003), internal or external changes that bring about new pleiotropic relations between various aspects of the organism along with new modularities. Modularity is therefore not in itself an aspect of either genes or any phenotypic trait, but is rather a contingent and context specific process by which genes can get dissociated from one another during ontogenic development. Thus, while anti-GMO activists may push the idea that Sewall Wright would call “universal pleiotropy” (Wright 1968) too far, arguing vacuously that “everything affects everything,” what is more accurate would be to say that the absence of pleiotropy is itself just a particular context-specific pleiotropic effect (perhaps owing to the compartmentalization of different cell types or the heterochrony of different life stages or polyphenisms) and not an ontological fact about the atomistic nature of the genetic information. A gene (like an organism in the case of ecosystems) can be isolated from its contexts for certain purposes but is not acknowledged to be an experimental artefact. Because the more reliable interventions are always going to involve those genes that appear to behave discretely and independently of context, the commercialization and normalization of genetic engineering reproduces the assumption that this experimental artefact is an actual entity (a “natural kind” (Quine 1969)), leading humans to more deeply believe that the universe is comprised of things operating in relative isolation rather than in contingent co-evolution. Seeing genes as entities operating in isolation with preset functions is therefore a case of a more general type of thinking from which it emerged, but that it in turn solidifies. It buttresses with analogous ontological reductions, such as ignoring of interconnectedness in ecosystems and treating species in isolation. Each case is the product of assuming that the analytic method, which was developed as a tool to glean a relationship between empirical phenomena, is revealing an ontological distinction.

The logic associated with genetic reduction has other collateral effects on our mental ecology. One result is that genetic atomism further reifies the epistemological notion that other objects in the universe (such as humans) are fundamentally individuals. This interlocks with a number of political and social ideas associated with atomic individualism and leads our current globalizing culture away from a more nuanced and context-dependent understanding of the relationship between modularity and integration in social relationships. It does this by presenting an alleged example of an atomic individual, which functions as evidence of the atomistic metaphysical structure of phenomena in general. A further result is that genetic reductionism tends to prop up and shield the obsolete nature-nurture dichotomy against attack. When genes are described as information-bearing entities without a sensitive and ongoing responsiveness to environmental cues, the logic of the gene and the logic of the environment are artificially kept at distance from one another. Protecting this dichotomy is not without consequence as it itself is tied to frameworks rationalizing dominance and supremacy of certain people over others. A third result (and one emphasized by Kirschner and Gerhart 2005) is that genotypic novelty ends up getting described as “error” or “mutation” which becomes what philosophers call a “black box”, hiding the fact that the explanandum has been shrouded by a concept that does not clarify it in any way. If the genome is sensitively reactive to a multitude of factors from within and outside the cell, novelty is explained through the new configurations that the system can undergo. Not having an adequate explanation of novelty gives creationists and intelligent design theorists a soft spot to jab that they ought not have (see Griffiths and Stotz 2013). Finally, the assumptions underlying genetic reductionism are leading to an increased interest in sequencing DNA in order to predict health and disease, ignoring the fact that correlations only have statistical validity made possible by relatively stable environmental traits (Hubbard and Lewontin 1996).

Another reified disanalogy is the assumption that genetic engineering is mimicking and is therefore fundamentally similar to the process by which nature generates variability for selection. Many evolutionary biologists are increasingly adamant that it is the developing phenotype perturbed by, and actively re-adapting to, environmental factors that is the most evolutionarily significant source of novelty (Kirschner and Gerhart 2005; West-Eberhard 2003). An environmental change provokes responses in organisms that are themselves variable, and whose plastic responses have varying likelihoods of being favoured by selection. This has lead West-Eberhard to state that “genes are followers in evolution” (2003: 157) and that the gradualist adaptationist assumption that sees favourable genes slowly make their way through a population is rarely powerful enough to have evolutionary significance. The gradualist paradigm and the logic of genetic engineering interlock to normalize the notion that causality is bottom-up and non-contextual and that the organism’s contribution is epiphenomenal. As readers of this journal know, such a position is no longer warranted (for the active role of semiotic agents in evolution, see in particular Hoffmeyer and Kull 2003; Sharov et al. 2016; Sharov 2016).

Of course, observed unanticipated side effects temper the notion of genetic reductionism.^12^ However, unless the side effects are catastrophic, they are likely to be interpreted as “exceptions” to the predominantly modular nature of the genome.

## Further Normalizing Eugenic Thinking and Practices

The self-validation of eugenic thinking is another obvious consequence of the normalization of genetic engineering and associated biotechnologies. Eugenics, or the science of improving the race of a species, was developed as a concept by Darwin’s cousin, Francis Galton (1904). The complex history of eugenics cannot be treated in this article. It was fairly mainstream thinking amongst Western intellectuals and politicians in the first few decades of the twentieth century but became offensive (at least as regards human eugenics) once Nazi Germany began institutionalizing its practice. Although many consider eugenic-type concerns with genetic engineering as absurd or inappropriate, this is a dimension that we should take seriously in the development of human mental ecology.

The purpose of GMO research is to improve the species modified, where “to improve” is taken to mean to change its traits in a way that is seen as desirable to humans.^13^ To “improve” assumes that the human is able and entitled to identify what traits are better augmented, added, attenuated, or erased in an organism. This assumption cannot be simply accepted without consideration of self-validating effects. It needs to be *warranted* especially considering many of the features which we consider to be an “improvement” are based on very short term concerns based on significant limitations in understanding. We are still discovering what genetic factors are needed in an organism to ensure long-term viability (for either it or us, or other species dependent upon it). There are therefore immediate ecological and evolutionary concerns. Eugenics is problematic not simply because it led to murderous implementation but because it raises the broader philosophical question as to whether people have the right (let alone capacity) to decide what is a “better” or a “worse” human and to control the process thereby. A rejection of eugenic thinking means a deeper acknowledgement of the fact that evolution is much more embracing of novelty emerging as the creative (stochastic) element of the process, in contradiction to the narrow and vicious characterization of a red tooth and claw world. Eugenic thinking is supported by the Modern Synthesis’ approach to evolutionary theory, which emphasizes the process of selection rather than the process of variation (West-Eberhard 2003). However, this approach is compatible with a goal-centred attitude toward evolution: instead of fostering the conditions that ensure the spontaneous production of diversity in biological systems, effort is placed on trying to control outcomes for ends imposed on the system by the “selector.”

It may be argued that traditional plant breeding is eugenic as well, and it certainly is – at least halfway. A major difference, at least from the semiotic view taken in this paper, is that the creative step is still made “by” nature (i.e. we do not control what traits emerge (even with extreme methods such as mutagenesis), we only select which ones we desire to keep). With genetic engineering, eugenics is more comprehensive because it includes both the engineering and development of the trait and the selection of the organism expressing it. For this reason, it magnifies an approach to valuing and rating other species better tempered than kindled. Our limited understanding of ecological networks suggests that we take a magnanimous attitude towards genes, species, and diversity, with the burden of proof placed on whoever insists that the interconnected system can be safely reorganized. In general, we need to avoid using past patterns of thought as precedents for allowing more extreme versions of that thought if and when it leads to consequences that we do not desire. In other words, because ecological studies would suggest the importance of a mental ecology that perpetuates humility in the face of complex systems that we depend upon for survival, we must be vigilant about safeguarding against feedback loops propagating the idea that humans know what is better in such systems. We need to build into our mental ecology ideas that can govern and prevent destructive ideas from taking over the conceptual landscape, and put them in a healthful balance. This will be discussed more thoroughly in the final section on educational implications.

The fact that the National Academy of Sciences in the United States recently published a report recommending human germ-line engineering suggests that humanity needs to urgently and publically discuss the semiotic effects of engineering humans. Cosmetic interventions are currently allowed in medicine, and there is no reason to think that a sharp line between essential and inessential human engineering is easy to demarcate or police once the normalization process is underway.^14^ While we do have some important warning stories in the mainstream (like the movie *Gattaca*, dir. A. Niccol 1997), the fact that eugenics is now widespread in other plants and animals means that it is only time before the boundary between animals and humans is broached on a broad scale. In other words, we have already become acclimatized to a mental ecology that normalizes eugenics as a way of seeing and redirecting evolution. Because humans are not different “in kind” from other species, incremental habituation in thinking jeopardizes the force of arguments against eugenic approaches to improving the human germ-line (Loftis 2005).

A related concern is that the spread of genetic engineering also further propagates and encourages an exploitative and resource-extractive conception of other species. What opens up is a view of life where it is seen as a part of the machinery by which market-based economies reify the system of ideas that sustains them at the expense of the mental ecologies needed to integrate and co-evolve in the biosphere. When we engineer cows to produce pharmaceuticals in their milk we normalize the idea that an improved cow is not one adapted to better achieve its own potentialities (which was arguably at least a part of Galton’s vision) but one that has been reconfigured in order to function better in the production and extraction of resources for humans. The biocapitalist dimension of biotechnology has been explored thoroughly so I will not repeat it here (Shiva 1997; Pierce 2013). Once genetically engineering humans takes place, it will occur in an economic context that will benefit those with the means to use it, potentially entrenching and exacerbating existing inequalities. If engineering does confer advantage, inequality founded on problematic socioeconomic conditions would become biologically inscribed.

## Relocating the Source of Creativity to Human Innovation

Increasingly, Bateson came to believe that the health of our mental ecology depends on practices that enable us to experience that we are parts of systems much larger, more powerful, and more complex than is consciously perceived. He thought that most religions were “true” in this way: their beliefs led to pathology error correction even if their particular premises were scientifically nonsensical (Bateson and Bateson 2004). To be clear, Bateson’s purpose was not to rekindle some twenty-first century version of a prior theism. He was a scientist through and through. It was rather to acknowledge that people can only ever have a partial and situated understanding of the large, complex processes that give rise to living ecologies – and that acknowledging this fact is itself a necessary strategy to help avoid some of the destructive consequences inherent in the type of intelligence we have.

The point of this section is to argue that by engineering the most complex things in our environment (i.e. living systems), humans risk losing some of the last mental checks available against runaway hubris. Our species has been engineering inanimate things for a long time and in many cases has added complexity to them (like when builders turn iron ore into a bridge). Nevertheless, engineering has, until recently been limited to creatively re-organizing things without much internal organization of their own. The most complex and creative processes in the universe were left untouched and therefore so was the capacity to appreciate the fact that such complex systems exist. A reader of a draft of this article commented that I ought not anthropomorphize the term “creativity” in this discussion. This concern only shows how far Western societies have succeeded in stripping nature of any adjectival dimensions that acknowledge its ongoing generative potency. The fact that using the word “creativity” to describe natural processes is no longer allowed is part of the mental ecology that situates humans as demiurges in a world there for manipulation.

The justification for transferring creativity to the human is packed into prevalent assumptions about the source of novelty in genetic evolution. As mentioned above, our understanding of random mutation and the defensibility of genetic engineering are mutually associated. Let’s explore this further. On the one hand, the randomness of the process implies that genetic engineering is simply introducing one more source of variation ultimately to be culled or supported by the process of selection. On the other, the fact that genetic engineering sometimes works appears as proof that mutation really is random. These concepts couple to thwart our capacity to recognize both the incompleteness of our concept of randomness and the potential risks of genetic modifications. Not all parts of the DNA mutate at the same rate, some parts are highly conserved, other parts highly mutable (i.e. mutation “hotspots”), implying that the relative capacity of various parts of the genome to develop novelty has itself evolved (Benzer 1961). In addition, which parts are mutable is itself flexibly adaptive depending on context (Wright 2000, 2004). What these findings imply is that while the actual mutation may be undirected (i.e. “isotropic” – and there is some evidence against this long-held proposition (Shapiro 2011)), it is differentially enabled. Once enabled, it is also differentially maintained by how the cell repairs, eliminates, or regulates the change (Martincorena et al. 2012). Thus a directedness *does* appear in the consequent types of mutations that remain stable in the organism. So, when genetic engineering involves the silencing of these regulatory mechanisms in order to encourage “proper” expression of the transgene, the directed (i.e. non-random or the source of apparent teleology) part of the emergence of novelty in the organism has been tampered with. Genetic engineering thereby risks reifying the ontological assumption that mutation occurs without context and that the organism’s semiotic activity plays no role in the outcome, each which validates the acontextual randomness of the genetic intervention – or rather the assumption that the only true source of context is that provided by humans.^15^

Heidegger (1977) explored the phenomenological dimension of this essentially cybernetic fact in his description of how technologies conceal the *poeisis* of nature by organizing it so that it serve purposes set by humans. The technology then reveals the world as ordered by an anthropomorphic causality which subsequently informs the thinking of the human experiencing it. An increasingly technological world is continually re-established as the new normal by those living within it, entrenching means-end rationality as the mode by which phenomena in the world come to appear. In the case of genetic engineering, the organism has not simply become the “standing reserve” Heidegger feared as exemplary of the dangerous mutual ordering of technology and thinking.^16^ Indeed, the mental reconstruction of our understanding of life, such that we see its form, its physiology and its behaviour as now parts moulded and recircuited, represents a much more significant form of ordering than Heidegger imagined. Biology, the study of life, has transformed into the study of exploitable biological information, a shift ripe with ethical and political consequences (Pandilovski 2012). Under such a paradigm, it can now be proposed – unfacetiously – that humans engineer animals for ethical reasons, exemplified in the suggestion that factory-farmed animals be re-ordered to no longer suffer from our ordering impositions: “would it not be positively right to breed, or engineer, [a chicken] as a deaf, blind, featherless, legless, anencephalic lump that we could not injure further?” (Clark 1998: 217). Further, biotechnology is based on the reconstruction of life, but because it is concerned with modifying heredity, ultimately evolution. The massive spatial presence of the means-end conversion of a river into a hydroelectric dam that so haunted Heidegger has now found a rival in the even more massive temporal presence of diverting the evolutionary processes of life by means-end thinking, with future effects as boggling as the scales of time it took to get life to the stage it is at today.

Problems are identified by industry, which then develops the technologies to solve them. The rate and pace by which ecological systems “solve” problems is eclipsed by the rate of change of industrial research and development. Beyond the food crops that form the basis of activists’ pitted attacks, many wild species are engineered in labs for possible release into the wild, including mosquitoes, forest trees, etc. As the biological diversity of ecosystems plummets, bioethicists debate whether society is morally obligated to create biological diversity to replace what has been lost (Boldt 2013), a position embodied in Brand (02013)^17^ vision of bringing back wooly mammoths and other species decimated since the dawn of the Pleistocene.

Educators need to be pre-emptive against the possibility that innovation and complexity in the biological world is shadowed by the creativity of humans, because the process is at risk of Batesonian self-validation. Biotech inventions are foregrounded, natural “inventions” go extinct; the small genetic changes “we” make are highlighted, the larger genomic, cellular, and ecological contexts that our experiments are dependent upon are backgrounded. If we agree that humanity needs to experience the creative and spontaneous self-arising of a process that is bigger than us (Bonnett 2016), then we should pause and consider the experiential dimension quietly silenced under the cloak of human purpose.

Consider the recent “Glowing Plants” project on Kickstarter,^18^ intending to engineer glow-in-the-dark trees to replace street lamps. People would see such a tree and be dazzled by the ingenuity of the scientist, clouding the fact that the phenotypic changes experienced are themselves natural inventions (i.e. humans did not create the firefly gene; horizontal gene transfer; the tree’s metabolism, semiotic activity, or transcription machinery; the capacity for retinas to be triggered by wave frequencies, and our brain to transform it into the experience of light; our ability to experience amazement, etc.). It then requires much pedagogical effort to uncover from underneath the human “bling” the complexity that humans remain dependent on in order to regain that sense of awe and humility before the world. This flies in the face of an educational approach more consonant with Bateson’s insights: that we are called upon to develop a sense of reverence for life through practices that nurture what Rud calls “a sense that there is something larger than a human being, accompanied by capacities for awe, respect, and shame” (Rud 2011: 55). Indeed science can be employed to uncover the profound ways in which humans are interconnected with and dependent upon the complex processes of the natural world. But it can also instrumentalize the natural world by turning it into machines and resources, making it appear as serving human purposes and therefore subordinate to our power. This latter capacity tends to be parasitic upon the former one indicating that humans must be ever-vigilant in questioning to what extent such an instrumentalization is necessary, as well as to what extent educators need to develop cultural practices that can compensate for this instrumentalization by foregrounding that which is still able to instigate a sense of reverence-worthy self-arising natural process. According to Bonnett (2016) this is what we must *sustain* and what must be integral to any concept of sustainability.

For Barnes and Dupré (2008), the sense that we are *mastering the master molecule* has given rise to both the excitement and the profound anxiety about what genetic engineering means for society and life on earth. They suggest that we de-hype the molecule. This seems like sage semiotic advice. But it does not seem like the hype is easy to overcome, considering it is entwined in multiple processes, identities, and economies. It is likely that the paradoxical tension is here to stay unless we explicitly engage with it. It is a pedagogical question how to frame genetic engineering. If educators conclude that mental ecologies require buffering of hubristic thinking, and that this can come about through acknowledging that we live in systems more complex than we conceive, then explicitly highlighting side effects of genetic engineering should be an educational priority (Affifi 2016). Such unintended consequences need to be brought to attention regardless of whether they are malignant or benign. Research that uncovers unintended consequences can come from omic studies,^19^ behavioural or physiological studies, and ecological studies, all which will reveal the novel products and interactions of genetic intervention on different biological and ecological scales. For example, a proteomic study of Monsanto’s MON 810 maize revealed that dozens of protein changes result from up or down regulation of other genes as a result of the inserted genes (Zolla et al. 2008). These changes are not likely dangerous, but they were not predicted or desired. The problem is that it currently does not serve industry to communicate the extent of benign side effects so such studies are often claimed to be unnecessary or misleading (Lay and Liyanage 2006).

Humans are faced with a dilemma. If the uncertainty, pleiotropy, context-specificity, and semiotic activity of the process is acknowledged, we save ourselves from an undesirable mental ecology that comes from living in a world that we believe is genetically controllable. The indeterministic creative aspect of the complex system is foregrounded. However, what is lost is assurance that the process is definitely safe or well-understood. For industry, the pedagogical choice is obvious: given that biotechnology depends on grants and stock shares, the industry will not likely seek to protect this sense that even the engineered system is “larger than we are.” They will probably continue to develop GMOs based on genes that have less visible side effects and will continue opting for the irrelevance of studies that would reveal the unexpected dimensions of intervention. Funding for and marketing of products requires entrenching the impression of a successfully controlled and manipulated nature.^20^ However, educators need to recognize that the way in which genetic engineering discourse emphasizes the concept of control while silencing the empirical significance of uncertainty has much bigger semiotic impacts than merely ensuring the economic success of a company. A transparent and comprehensive exploration of the side effects of genetic engineering on all scales, from the biochemical to the ecological, but also its semiotic dimensions within human mental ecologies, would emphasize uncertainty, protect from the runaway effects of hubris, and thereby ensure that those genetic interventions society decides to pursue are not taken lightly.

********

I have said that these four factors are interlocking, but I will not show all the possible interrelations between them. One could say that these four factors are themselves *pleiotropic*, and are more or less separable as factors depending on the actual sociocultural conditions that occur and differentiate them. However, I will name a few linkages. I think it is easy to see that the habituation to exposure of GMOs lessens our general concern the logic that sustains their continued development, that part of this logic is eugenic reasoning and a reductionistic abstraction of the function of genes, and that all these processes are further stimulated by our reduced acquaintance with the creativity and generativity of nonhuman (semiotic) processes, which are in turn camouflaged by the rearrangements of our mental and physical environments that genetic engineering progressively affords. The process works in reverse too. Producing technologies based on the assumptions of genetic reductionism stimulate the idea that its logic is correct, which itself stokes hubris by positioning humans more confidently as the knowing and able exploiters of the universe’s intricate secrets. As the complex interconnections between the genome and cellular and environmental factors face a diminishing role in the production of novelty, humanity is further entrenched as that factor responsible for redirecting the process for eugenic improvement all the while circumventing the need to understand these very processes.

On the other hand, it is entirely possible that these progressively mutually reinforcing feedback loops collapse. Indeed, it is the nature of positive feedback loops that we should anticipate this at some time. Genetic engineering could lead to a significant catastrophe that casts doubt on the patterns of thought and behaviour connected with it. Controversial risk analysts Taleb et al. (2014) suggest that even if current safety regulations ensure it vastly unlikely that any single release of a GMO will cause significant ecological harm, the continued release of an increasing number of GMOs magnifies greatly the likelihood that at some point a large-scale event leading to regional or global harm will occur. As pointed out earlier, if these events are not catastrophic, unanticipated side effects will cause temporary doubt in the biotech project and may add reactionary oversight to the regulation process but the momentum of the industry and its integration into society will likely mean that we continue betting against the probability of a more serious future calamity. On the other hand, if the consequences lead to non-recoverable (at least on any normal human scale) ecological ruin, as these authors predict is plausible, we would likely undergo a serious re-evaluation. The destabilizing effects on ecosystems might become severe enough that they lead to a correction of the pathological mental ecology that brought them about, to a realization and felt awareness of that “something bigger” that Rud described as so key to a sense of reverence. In this way, the temporary runaway destruction in biological systems would trigger a negative feedback effect balancing our hubris. However, this extreme case would cause much suffering and impoverishment to human and other worlds. It is outside of pedagogical consideration however much it eventually proved “educative” in terms of improving our ontological assumptions about our place in the scheme of things.

## Implications for Educators

Throughout this article, I have assumed that readers of this journal are all explicitly or implicitly science educators and I have made some suggestions for helping understand the consequences of genetic engineering as a way of thinking. In this section, I will summarize and expand on these suggestions. The suggestions outlined here are based on the assumption that unless educators develop specific strategies to limit the types of thinking normalized through living in a world composed through genetic engineering, society will be unable to make decisions appropriate for avoiding some of this trajectory’s undesirable consequences. The goal of education here is to develop ways of thinking that work to interfere or limit the growth of 1) the engineering metaphor as applied to life, 2) the concept of programming organisms, 3) the concept of “improving” living organisms, including humans, and 4) the relocation and concentration of creativity within human activity. There are undoubtedly other types of thinking normalized through these technologies deserving further study.

Educational implications can be divided into goals and objectives for educators. The overall educational goal is to ensure that society more deeply understands the actual and potential impacts of genetic engineering and is able to use this understanding to critically, responsively, and democratically decide on the role which genetic engineering takes. The actual and potential impacts, this paper argues, are not limited to direct effects on health or the biosphere, but also involve semiotic effects within the ecology of ideas that contextualize human thought and action (and which may then lead to health or biospheric effects). In order to meet this educational goal, specific objectives depend upon the extent to which genetic engineering has already come to affect human mental ecologies. If people are already habituated to the notions described in this paper, then different pedagogical approaches will be needed than would be the case were educators instead seeking to be preemptive against habituation to ideas not yet normalized. I shall address these specific challenges in a moment, but will first of all point out that in both cases it seems that a better understanding of the behaviour of genetic engineering ideas in human mental ecology is necessary, prompting the need to foster general public understanding of 1) the nature of genetic engineering ideas, and 2), the ecological nature of thought, including how thought processes constitute and constituted by the material worlds humans inhabit.

Understanding the particular effects of genetic engineering ideas on mental ecology requires a better understanding of the limitations inherent in the engineering, programming, and reductionistic metaphors; a better understanding of the nature of the genes and genetic information in light of widespread advances in genomics and the emerging paradigm which understands information as constituted by complex mental interactions between genes, organisms, and environments.

Understanding the ecological nature of thought requires increased familiarity with basic ecological ideas such as positive and negative feedback, and hierarchical governance of feedback by higher order feedback. Some areas of the biological sciences are already modelled in these ways, so it would make sense for educators to help students familiarize with ecological phenomena in these fields first. Many examples are available in biology textbooks, such as the circularity of the carbon cycle and the hierarchical governance of genes by other genes (such as the standard example of Hox genes illustrates). However, biology textbooks just as often present linear slices of circular systems, and do not emphasize that circularity is an inherent and necessary aspect of the causality of living systems. For this reason, educators need to constantly emphasize that linear representations of living systems are isolated for pragmatic purposes and are not representative of the causal nature of the phenomena. Once the capacity to perceive and understand circular systems has become itself a habit of thought, it will be easier to apply this type of thinking to understanding the slightly more abstract cases of ecological relations amongst ideas and systems of circulating differences. At this point, it is beneficial to cultivate a self-reflective understanding of how these feedback relations exist within human mental activity and not just in external biological ecosystems, a general understanding of how these mental ecologies affect and are affected by environments, technologies, and the like, and finally, an understanding that cultures have developed ways of tempering potentially destructive positive feedback in their own mental ecologies through cultivating higher order belief structures such as those we find in religion.

Our educational challenge is then to help people understand more specifically how ideas about genetic engineering interact in mental ecologies, and this paper has attempted a preliminary tracing of how such ideas may interact. Simply *knowing* such interactions is, however, in itself of little value. The point is to understand the actual and potential consequences of these interactions, to redflag those we do not desire, and to make interventions into these mental ecologies to sidestep side effects. This is inherently a pedagogical activity as it involves consciously taking responsibility for how such ideas interact. On the one hand, the educator is then tasked with assisting students in developing skills by which to take responsibility for and transform their mental ecologies such that they may be more sustainable. On the other hand, the educator, as one with a greater awareness of such effects and the tools by which to avert them, is also responsible for curating experiences that can assist students in avoiding detrimental effects while students are in the process of developing the skills to avoid them on their own. It is in this latter context that educators have to deal with different cases depending on the extent to which genetic engineering ideas have incorporated themselves into the mental ecologies of their students.

In the case that such ideas are new and only beginning to expand in students’ ecologies of ideas, a number of concrete strategies can be adopted. First of all, we can limit our exposure to organisms we consider to be engineered. Even though many parents currently limit their children’s access to violence on television because they are aware that violence may become normalized, we do not often approach new technologies with an eye on how the metaphors that they reify could be affecting both adults and children. An obvious objection to this suggestion however is that it reproduces the same problem as hidden clearcuts, slaughterhouses, and other practices that activists work to make more transparent and is therefore potentially a regressive suggestion.

An alternative approach would be to expose where and how the engineering metaphor (and the concepts it seems to depend on) does not work. The transgenic or gene edited organism has not been deterministically reorganized, complex uncertainties are still there, and “nature” (as in that which is not imposed teleologically by humans) is still present even throughout these modifications. As noted, industry has reasons for limiting public awareness of such uncertainties even when they are not likely harmful for the environment of human health. However, pedagogically, it is likely necessary to bite the bullet and risk increasing the public’s apprehension of such technologies in order to eventually arrive at a place where the decisions if or when to genetically engineer are made with care and foresight.

A third strategy is to make sure that the presence and nature of wild organisms is accentuated in different ways in students’ experience. On its own, this strategy is likely insufficient as well, unless it is accompanied by experiential and theoretical work that can open people to appreciating the natural world in deeper ways. This is because while an engineering mentality may not pervade students’ attitudes towards other species, their mental ecologies may still be interlocking a prior set of limiting ideas about what nature is and is capable of. In particular, providing educational experiences that draw out organisms as active interpretive agencies is seen as crucial.

A fourth strategy would be to reconstruct the language used when speaking about and engaging in genetic engineering.

A fifth strategy might be to foster types of ideas that can help keep in check ideas that would become deleterious if allowed to go into runaway. Bateson noted that healthy ecologies have positive feedback loops and that while these are important for the generativity of the system, it is also necessary that they be governed by other loops. An instrumental or an atomistic attitude towards other organisms may sometimes be necessary but we need a set of ways of undercutting this way of thinking once it starts to dominate the mental ecology. Bateson suggests that such higher order regulation traditionally came from religion but that a contemporary version could come from ecology and cybernetics. He also insisted that art was capable of integrating consciousness into the larger cybernetic mind (that it keeps forgetting it is only a component of), and is therefore necessary for fostering a visceral awareness of the limitations that consciousness has in creative process.

## Footnotes

1 With the advent of gene editing techniques such as CRISPR cas9, there is considerably more momentum and it is unclear whether traditional forms of activism will continue to have a consequential impact on commercialization rates.

2 I consider all readers of this journal to be science educators in some loose sense. Most are in academia and work with students. Those who do not are still engaged in multiple informal learning relationships in their daily lives within which values and attitudes about science are constantly implicit. The fact that the distinction between educator and non-educator has been institutionalized does not change the fact that there are pervasive educative dimensions to sociocultural experience.

3 Bateson suggests that “consciousness” occurs only in mental systems that become aware of the ‘product’ of their perception without being aware of the process that led to that perceptual product. It is by nature *fragmentary* because the conscious experience does not have access to the entire mental circuit in which it is dependent and embedded, and as a result it tends to systematically distort its understanding of how its own mental system is organized. For example, because of this fragmentary understanding, conscious organisms tend to interpret themselves as causal agents in a simplistically linear way because that is how their experience is disclosed to them. One point of this article is to show that the appearance of causality in genetically engineered organisms perpetuates this linear conception of mind, which is a misleading product of consciousness.

4 Of course, some of the patterns identified here are not solely the product of genetic engineering, but of the general system of ideas that facilitated the emergence of genetic engineering – which it then serves to further entrench. This dimension will be discussed in what follows.

5 Meanwhile, risk assessment methods, rigor and objectivity are biased by habituation. This phenomenon has occurred with many technologies in the past. Once habituation occurs, only a disaster is likely to respark initial concerns (as with the response to Fukushima), − and even then it is likely to be schematized as an exceptional event that ultimately does not shake the logic or rationale behind the continued deployment of the technology. Humans are much less capable of seeing how the things they are immersed in affect them and this psychological fact should be put at the forefront of any risk assessment so that people can explicitly correct for this automated bias when designing methods to evaluate and take precautions for new and existing technologies.

6 Whether or not ecosystems can support such radically discontinuous shifts in rates of change is a serious question. Organisms expect patterns of behaviour, and all evolution so far has been co-evolution. It is not clear that a breach in co-evolutionary processes is ecologically stable (see Wills (2001) for a discussion of the disrupting effects of genetic engineering on evolutionary processes). According to some models, at some point natural selection itself breaks down when the rate of novelty generation passes a threshold above which selection becomes impossible (Nowak and Schuster 1989).

7 Ignoring for the moment that corporations actively work to conflate processes to generate product acceptance amongst the wary.

8 Debate remains about whether it is still valuable pragmatically as a way of thinking about biotechnology research (Barnes and Dupré 2008).

9 William Bateson (1909) coined the term “epistatic” to refer to the process whereby one gene on a chromosome masks or silences the effect of another. The term “epistasis” has since broadened to describe any interaction whereby two or more genes contribute to a trait. Pleiotropy was a concept originally developed by Ludwig Plate (1910) to describe the phenomena whereby a single gene contributes to more than one trait. Both processes were considered uncommon during much of the Modern Synthesis (although Mayr (1963) and Wright (1968) both considered pleiotropy to be prevalent). Both are now known considered to be closer to the rule than the exception.

10 For example, certain signals cause chromatin (the DNA-protein chain) to bend, altering the type or rate of interactions that occur between its parts (Baker 2011). The chromatin twists and turns constantly, sometimes folding in upon itself to close off genes so that they cannot be expressed, and sometimes putting ones that are quite far away from one another into contact. Whether or how a gene can be said to be “regulatory,” i.e. influencing the function of other genes, is itself shaped by such environmental and developmental contingencies, so the distinction between regulatory and structural genes is itself context dependent. The behaviour of genes are steered by the cellular and organismal context that they are found in. Information and control are distributed and modified by the organism’s ongoing adaptive activities. Evidence of Batesonian mindedness is also seen in alternative splicing (Bradbury 2005; Wu et al. 2004). Once a gene has been transcribed into RNA, it undergoes an editing process that cuts out certain segments of it. What is left over is called mRNA (or messenger RNA) which is then translated into polypeptides (chains of protein) by the ribosome. Gene expression would be quite machine-like and uninteresting if it was predictable which elements are taken out during this editing process. But, in fact, about half of human genes are capable of producing RNA which can be edited in numerous ways, and sometimes (such as in neural cells) in thousands of different ways. What this means is that a single gene is capable of being expressed in thousands of different ways. What decides how it is being expressed is, again, the larger evolving context of the cell it is in, which includes signal transductions coming from within and outside the cell.

11 Hence Bateson scholar Harries-Jones (2016) describes the human genome project (and this obviously also therefore implies other genome projects as well) as one of the most extravagant wastes of money in the history of scientific research.

12 The extent and scope of side effects is difficult to assess in our current research context. Innovation is coupled with the particular socioeconomic conditions that bring it forth. In our case, these technologies are brought about in and stimulated by intellectual patenting laws and protections that celebrate and incentivize invention. This aggravates not only the pride and further motivation of the inventor but also fractionates the knowledge generation process so that the side effects of genetic engineering become less widely shared and theoretically synthesized. As each researcher or business develops its own confidential approaches, the results of their discoveries (and mistakes) are hidden from others through mechanisms erected to protect competitive advantage. The result is that partial and distorted understandings emerge, scattered across the globe, with very little scientific integration that cross-talk between the researchers would surely facilitate in a different economic context.

13 Of course, “improve” can sometimes mean to deliberately make something less strong, or able or independent from a biological point of view. See Newman’s (2013) discussion of meiogenics.

14 A major reason for the NAS recommendation was that the United States could not be left behind seeing as China and Sweden are going ahead with state funding of human engineering anyway (Harmon 2017). If China decides to proceed with research in engineering humans for inessential traits, like increased intelligence or physical function, it is difficult to imagine that competing countries will not now see such a research agenda as now “essential”.

15 Wills (2004) notes that the spatiotemporal context of genes and of organisms is crucial in Maori culture. He argues that the genes are located in organisms within a particular ecology and at a particular historical moment in a genealogy, and that there is an intrinsic value in this situatedness insofar as it manifests the co-evolving relatedness of biological processes and their co-dependence for survival. When we indiscriminately “invade natural genealogies we have started reducing the ordered tree of life to a twisted heap of broken twigs.” Wills therefore believes that genetically engineered organisms should be confined to laboratory conditions.

16 See Heidegger’s (1977) discussion of the conversion of a river’s water into a standing reserve through the development of hydroelectricity.

17 Date in a format Brand would approve of.

18 Visit their project website here: http://www.glowingplant.com/

19 “Omic” refers to a set of ways of analyzing gene products (Heinemann et al. (2011). It includes transcriptomics (the analysis of the RNA transcripts), proteomics (the analysis of protein products), and metabolomics (the analysis of metabolites). Omic studies can give an exhaustive list of such products at a given point in the organism’s ongoing development, which can then be compared with results from a wild organism at a similar developmental stage and environmental circumstance.

20 While the attempt to control heredity underpins commericalization, an opposite undercurrent is found in pure research. As synthetic biology and the artificial life research program move towards creating life, it has been observed that the design challenge is now to build something that is capable of novelty and spontaneity, which are seen as key attributes of living systems (Dupuy 2013). The irony here is that our tinkering with other organisms’ genomes is motivated by a desire for precision (for the organism to respond to the interventions in deterministic ways), while the aim of artificial life is to take what we think of as (more or less) deterministic inanimate matter and breathe indeterminacy into it. Success would be measured by the extent to which the engineered organism resists the application of the word “engineered”.
